# Physicochemical characterization of carbamylated human serum albumin: an *in vitro* study

**DOI:** 10.1039/c9ra05875c

**Published:** 2019-11-11

**Authors:** Asim Badar, Zarina Arif, Shireen Naaz Islam, Khursheed Alam

**Affiliations:** Faculty of Medicine, Department of Biochemistry, Jawaharlal Nehru Medical College, Aligarh Muslim University Aligarh Uttar Pradesh India kalam786@rediffmail.com

## Abstract

Carbamylation is an ubiquitous process in which cyanate (OCN^−^) reacts with the N-terminal amino or ε-amino moiety and generates α-carbamyl amino acids and ε-carbamyl-lysine (homocitrulline). The process leads to irreversible changes in protein charge, structure and function. In this study, we have investigated the effect of carbamyl (generated from potassium cyanate) on human serum albumin (HSA) structure and function. The carbamylated-HSA (c-HSA) showed various modifications when examined by UV, fluorescence, FT-IR and far-UV CD spectroscopies. c-HSA exhibited hypochromicity, loss in α-helical content, changes in the amide I and amide II band, *etc.* Native-PAGE showed increase in the mobility of c-HSA compared to native-HSA. Aggregate(s) formation in c-HSA was detected by thioflavin T dye. The biochemical investigations carried out on c-HSA suggested increase in carbonyl content and decreased binding of TNBS (trinitrobenzenesulphonic acid) and Sakaguchi reagent. The attachment of the carbamyl moiety to HSA was confirmed from MALDI-TOF results. The functional defects in c-HSA were confirmed from the low binding of bilirubin. Taken together, carbamylation of albumin caused changes in the structural and functional properties of HSA. To the best of our knowledge, this is the first report on detailed biophysical characterization of carbamylated-HSA.

## Abstract 2

Carbamylation is an ubiquitous process in which cyanate (OCN^−^) reacts with the N-terminal amino or ε-amino moiety and generates α-carbamyl amino acids and ε-carbamyl-lysine (homocitrulline).

## Graphical abstract

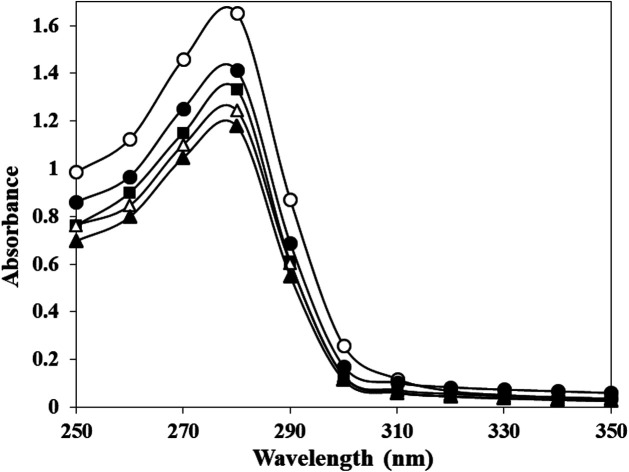

## 1. Introduction

Human serum albumin (HSA) is present in blood plasma. It constitutes about half of serum proteins. HSA transports hormones, free fatty acids, nitric oxide, poorly soluble drugs, bilirubin *etc.*, and maintains oncotic pressure. It contains three homologous domains *viz* I, II and III; each divided into two subdomains A and B and possesses 18 tyrosine, one tryptophan (Trp 214) and 35 cysteine residues; 34 cysteine residues are involved in intramolecular disulphide bonds and only one cysteine is free (Cys-34).^1^

Carbamylation (or carbamoylation) is an ubiquitous, non-enzymatic post-translational modification (PTM) which results from the binding of urea-derived cyanate. Earlier studies have suggested cyanate accumulation in patients with defective kidney function.^2^ In addition, myeloperoxidase (MPO) released from neutrophils and monocytes also produces isocyanate at the site of inflammation. PTMs are involved in various biological processes including enzyme activation, protein–protein interactions, protein transport *etc.*^3^ In addition, PTMs are also tracked as disease markers.^3^ Carbamylation basically refers to the addition of “carbamyl” moiety (–CONH_2_–) on proteins or amino acids.^4,5^ The concentration of isocyanic acid in the plasma of healthy subjects is ∼50 nmol L^−1^ but it can reach upto 150 nmol L^−1^ in patients with chronic kidney diseases.^6^ Two major sites of carbamylation reaction are, N^α^-amino moiety of a protein N-terminus and the N^ε^-amino moiety of proteins' lysine residues.^7^*In vitro* studies have shown that reactive oxygen species formed during carbamylation process may alter proteins' properties.^6^ Carbamylation plays significant role in progression of various diseases by altering proteins' charge, structure and function.^6^ For example, carbamylated-LDL possess atherogenic properties while carbamylation of erythropoietin leads to loss of erythropoietic activity.^6,8,9^ A major chemical effect of carbamylation is neutralization of positively charged lysines which changes protein–water interactions and alters ionic interactions on protein surface. Just as glycation contribute to pathologic sequelae in conditions such as diabetes mellitus, carbamylation has been shown to change the properties of various enzymes, hormones *etc.*, ultimately contributing to the deleterious effects of reduced kidney function.^9^ Carbamylation decelerates HSA function and carbamylated-HSA (c-HSA) is more prone to oxidative damage.^10^ Carbamylation derived products (CDPs) are highly reactive, heterogeneous class of compounds. Homocitrulline is a well characterized CDPs marker of protein carbamylation.^8^ In the present study, c-HSA has been thoroughly characterized by various biophysical and biochemical techniques and the effect of carbamylation on structure and function of HSA has been studied.

## 2. Material and methods

### 2.1. Materials

HSA (fatty acid free, 99%), thioflavin T, sodium azide and dialysis tubing were purchased from Sigma Chemical Company, St. Louis, MO, USA. Potassium cyanate (KCNO) and bilirubin were obtained from CDH chemicals, India. Sodium chloride, α-naphthol, sodium hypochlorite, sodium acetate, and sodium hydroxide were purchased from Qualigens, India. 2,4-Dinitrophenylhydrazine (DNPH) and TNBS (trinitrobenzenesulphonic acid) was obtained from SRL, India. All other chemicals were of analytical grade.

### 2.2. Carbamylation of HSA

HSA (0.045 mM) was mixed with potassium cyanate in the molar ratio (HSA : KCNO) of 1 : 555, 1 : 1111, 1 : 1666 and 1 : 2222 and incubated at 37 °C for 6 h in 150 mM phosphate buffer (pH 7.4). The unbound potassium cyanate was removed by extensive dialysis against double distilled water for 24 h at room temperature. Solution of HSA (0.045 mM) devoid of potassium cyanate and incubated under identical conditions served as control.

### 2.3. Spectroscopic analysis

The ultraviolet absorption profile of native and c-HSA was recorded on Shimadzu UV-1700 spectrophotometer in the wavelength range of 250–400 nm using quartz cuvette of 1 cm path length.^11^ Hypochromicity at 280 nm was calculated from the following equation:
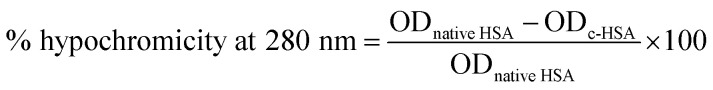


Fluorescence measurements were carried out on Shimadzu RF5301-PC spectrofluorometer at 25 ± 0.1 °C. Tryptophan fluorescence was obtained after exciting the samples at 295 nm and emission spectra were recorded in the range of 290–400 nm.^12^ Loss in fluorescence intensity (FI) was calculated from the following equation.
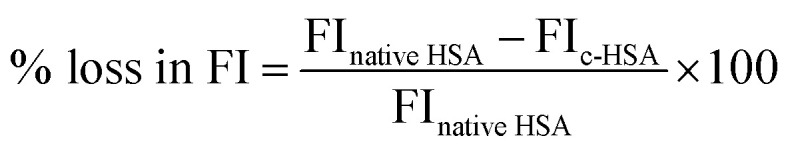


### 2.4. FT-IR and circular dichroism (CD) studies

FT-IR spectra of native and c-HSA were recorded in the range of 1800 to 1400 cm^−1^ in order to study the changes in the amide I and amide II band position.^13^ Very briefly, 10 μL of each sample was placed on the attenuated total reflection (ATR) unit of the machine and scans were obtained.

Far-UV CD spectra of native and c-HSA were recorded on spectropolarimeter. The instrument was calibrated with D-10-camphorsulfonic acid. The samples were placed in 1.0 nm pathlength cell and spectra were obtained in the wavelength range of 195–250 nm; scan speed 100 nm min^−1^; response time 1 s.^13^ The mean residual ellipticity (MRE), expressed in deg cm^2^ mol^−1^ was calculated from the following formula:
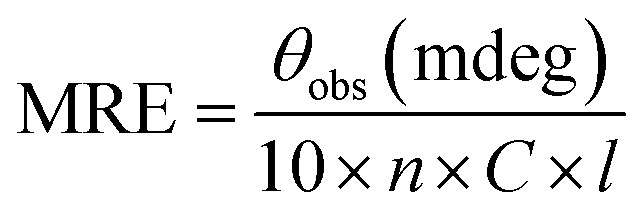
where, *θ*_obs_ is the CD in mdeg, *n* is the number of amino acid residues in human serum albumin (585), *l* is the path length of the cell in cm and *C* is the concentration of protein in mol L^−1^. The α-helix percentage was calculated using K2D3 software.

### 2.5. Gel electrophoresis

Electrophoresis of native and carbamylated albumin was performed on a 10% native polyacrylamide gel.^14^

### 2.6. Thioflavin T binding to native and c-HSA

Thioflavin T (Th T) is an authentic biochemical probe to detect aggregate(s) detection. Stock solution of Th T was prepared in double distilled water and an extinction coefficient of 36 000 M^−1^ cm^−1^ was used to determine concentration. Native and c-HSA was incubated with Th T (1 : 2) for 1 h at 25 °C. The emission fluorescence was recorded between 450 to 600 nm after excitation at 435 nm using an appropriate blank.^15^

### 2.7. Estimation of reactive carbonyl

Carbonyl content in native and c-HSA was determined using DNPH reagent.^16^ The absorbance was recorded at 360 nm against guanidinium chloride as blank. A molar extinction coefficient of 22 000 M^−1^ cm^−1^ was used to calculate the concentration in mol mol^−1^ protein.

### 2.8. Estimation of free lysine

ε-Amino groups in native and c-HSA were estimated using 2,4,6-trinitrobenezene sulphonic acid (TNBS) reagent.^17^ The protein samples were diluted to 3 × 10^−3^ mM in 100 mM sodium bicarbonate buffer (pH 8.5) followed by addition of 0.25 ml of 0.01% (w/v) TNBS solution and the reaction mixture was incubated at 37 °C for 2 h. After incubation, the samples were solubilized in 0.25 ml of 10% SDS; 0.1 ml 1 N HCl was added and absorbance was recorded at 340 nm.

### 2.9. Estimation of free arginine

Native and c-HSA was subjected to guanidium group estimation by Sakaguchi method.^18^ To 1 ml protein solution (0.045 mM), few drops each of 10% sodium hydroxide and α-naphthol was added. After mixing properly, 1 ml of 0.5% sodium hypochlorite solution was added. Appearance of red colour indicates the presence of substituted guanidines. The absorbance was read at 500 nm.

### 2.10. MALDI-TOF studies

MALDI measurements were performed on 4700 plus mass spectrometer (AB Sciex, MA, USA). The matrix was 50 mM sinapinic acid dissolved in acetonitrile/water (50 : 50, v/v). Native and c-HSA samples were diluted five times with 0.1% aqueous solution. Equal volumes of samples and matrix solution were mixed and approximately 1–2 μl of the mixture was deposited on multiprobe 384 well insert opti-TOF-stainless steel MALDI plate, air dried and the analysis was carried out with the help of protein chip software.^19,20^

### 2.11. Albumin–bilirubin interaction

Bilirubin (BR) interaction with native and c-HSA was studied by fluorescence. A stock solution of BR was prepared by dissolving 5 mg BR in 1 ml of 5 mM NaOH containing 1 mM EDTA. The stock was diluted with 0.06 M sodium phosphate buffer and concentration was determined spectrophotometrically using molar absorption coefficient of 47 500 M^−1^ cm^−1^ at 440 nm.^21^ All spectral measurements were made after 30 min of incubation at 25 °C in dark unless otherwise stated. Furthermore, the spectra were recorded under dim/yellow light to prevent photodegradation of BR.

The fluorometric titration of native and c-HSA with fixed amount of BR was carried out in a discontinuous manner. To a fixed concentration of c-HSA (0.003 mM) in different tubes a constant amount of BR was added and the final volume was made to 3 ml with 0.06 M sodium phosphate buffer. Emission spectra were obtained in the wavelength range 480–600 nm by exciting the BR-albumin complex at 460 nm.^22^

### 2.12. Stoichiometry of potassium cyanate–HSA interaction

The stoichiometry of interaction of KCNO and HSA was studied by absorbance measurement. The absorbance of 0.005 mM HSA was recorded at 280 nm in absence and presence of varying molar ratios of HSA and KCNO (1 : 1000 to 1 : 20 000). Job plot data obtained was plotted as change in absorbance at 280 nm as a function of molar ratios of ([KCNO]/[HSA]).

### 2.13. Statistical analysis

Statistical significance of the results was evaluated by Student's *t*-test and the *p* value of <0.05 was considered significant.

## 3. Results

### 3.1. UV-visible spectroscopy

**Fig. 1:**
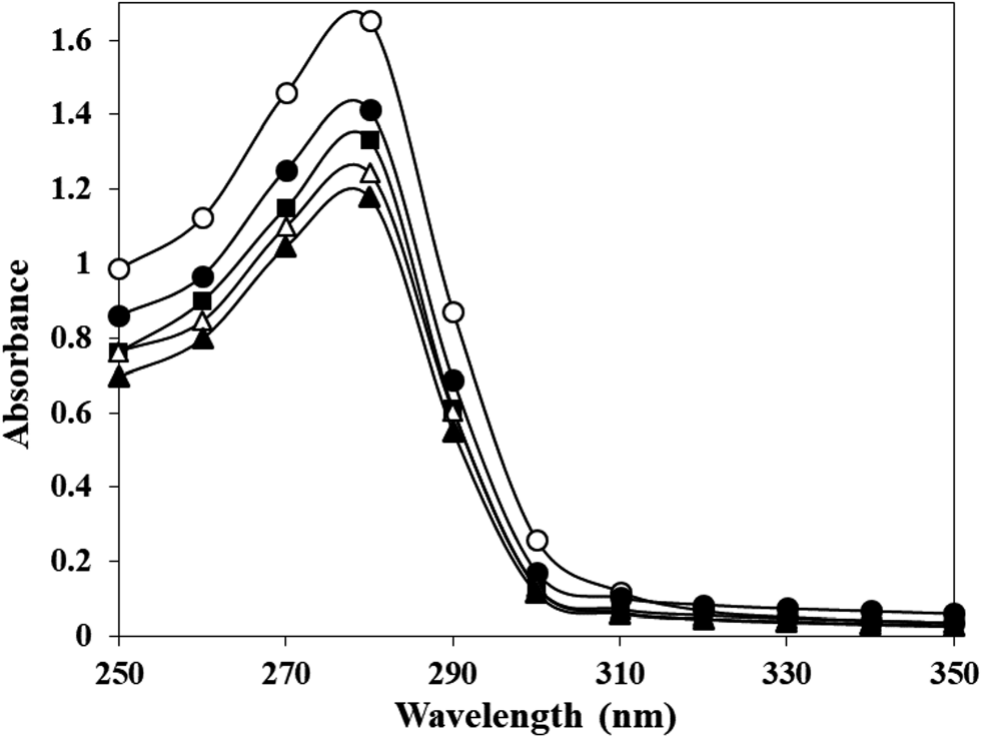
UV-vis spectra of native HSA (-○-) and at different molar ratios of HSA : KCNO, 1 : 555 (-●-), 1 : 1111 (-■-), 1 : 1666 (-△-) and 1 : 2222 (-▲-).

Human serum albumin modified with potassium cyanate in different molar ratios (HSA : KCNO ratio, 1 : 555, 1 : 1111, 1 : 1666 and 1 : 2222) for 6 h caused varying degree of hypochromicities at 280 nm (Fig. 1). The c-HSA showed 14.52, 19.30, 25.90, 28.63% hypochromicity at different molar ratios of HSA : KCNO as compared to native HSA.

### 3.2. Fluorescence spectroscopy

**Fig. 2:**
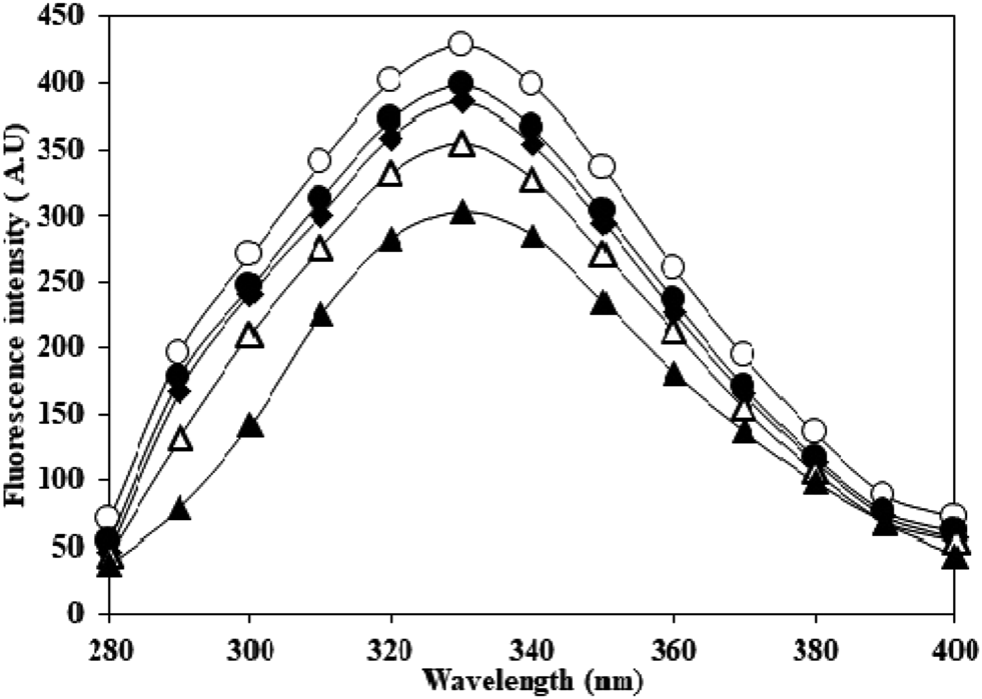
Emission profile of native HSA (-○-) and at different molar ratios of HSA : KCNO, 1 : 555 (-●-), 1 : 1111 (-♦-), 1 : 1666 (-△-) and 1 : 2222 (-▲-) excited at 295 nm.

Adduction of carbamyl group on HSA may also influence the properties of adjoining/neighbouring amino acids. This was probed by monitoring the emission intensity of tryptophan residues in native and c-HSA upon excitation at 295 nm. This produced quenching in the fluorescence which was directly proportional to potassium cyanate concentration. The results suggested that tryptophan residues contribute greatly to the quenching of intrinsic fluorescence of serum albumins (Fig. 2).

### 3.3. FT-IR and far-UV circular dichroism spectroscopy

**Fig. 3:**
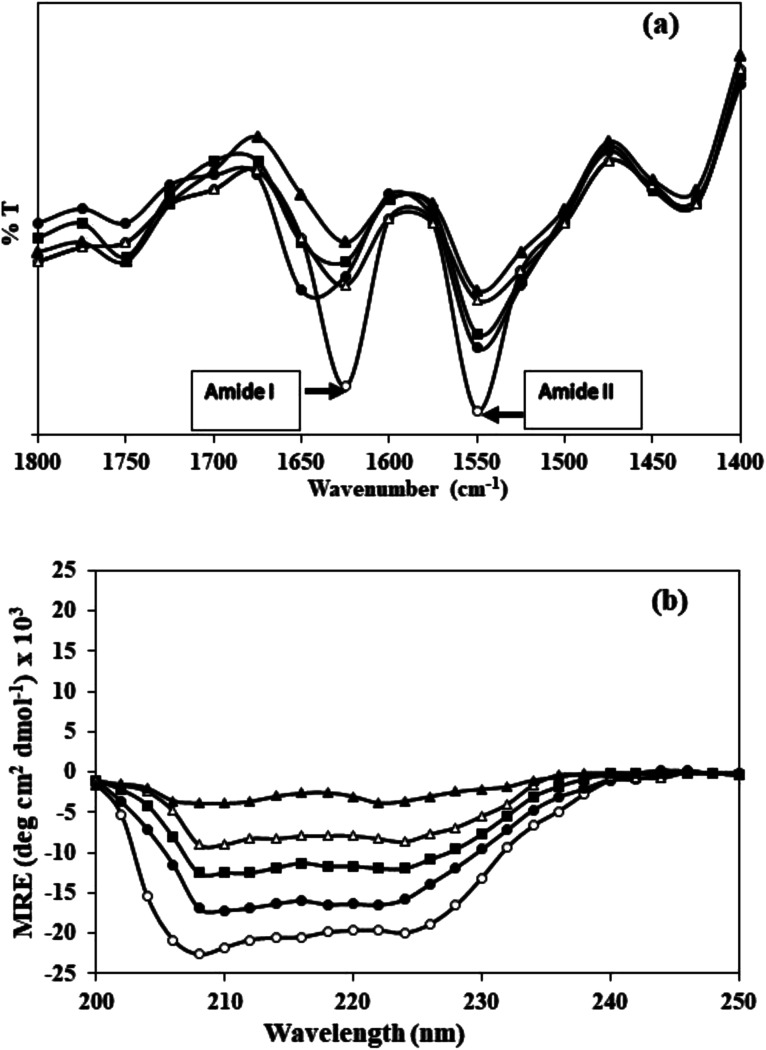
(a) FT-IR and (b) far-UV CD spectra of native HSA (-○-) and at different molar ratios of HSA : KCNO, 1 : 555 (-●-), 1 : 1111 (-■-), 1 : 1666 (-△-) and 1 : 2222 (-▲-).

Effect of carbamylation on the secondary structure of HSA was evaluated by FT-IR in the wavenumber range of 1400–1800 cm^−1^. The band positions of amide I (arising from C=O stretching) and amide II (originating from N–H bending vibrations of peptide groups and C–N stretching) in native HSA and c-HSA are shown in Fig. 3a. The decrease in the intensities of amide I and amide II bands in c-HSA samples as compared to native HSA speak in favour of changes in the secondary structure of carbamylated-HSA.

The results of FT-IR were further confirmed by far UV-CD study, which also suggested changes in the secondary structure of c-HSA. Far-UV CD of native HSA exhibited two peaks; one at 208 and another at 222 nm which is typical of α-helical protein. Native HSA possessed 68.1% α-helix which decreased to 67.99, 67.86, 66.4 and 23.21% when the HSA was carbamylated with different molar ratios of KCNO (1 : 555, 1 : 1111, 1 : 1666 and 1 : 2222) (Fig. 3b).

### 3.4. Native PAGE

**Fig. 4:**
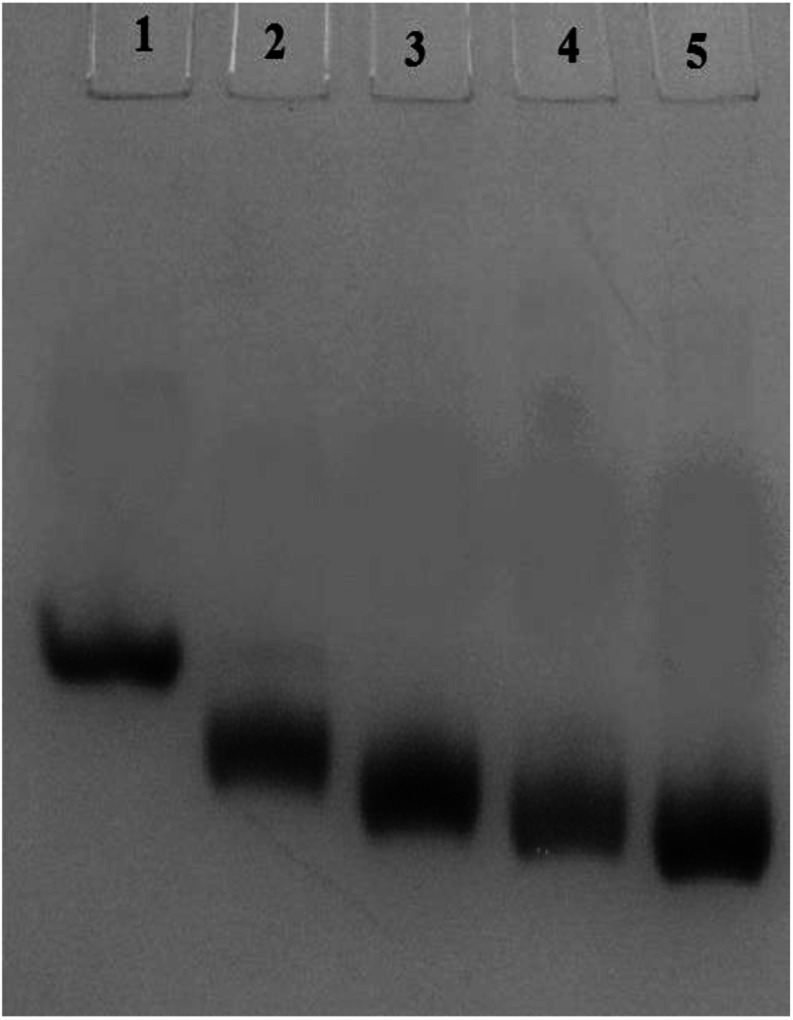
Electrophoretic mobility of HSA carbamylated with potassium cyanate. Lane 1 = native HSA; Lane 2 = 1 : 555 HSA : KCNO; Lane 3 = 1 : 1111 HSA : KCNO; Lane 4 = 1 : 1666 HSA : KCNO and Lane 5 = 1 : 2222 HSA : KCNO.

Carbamylation results in neutralization of the positive charge of the lysine and hence enhances the mobility of the c-HSA in native-PAGE. In our experiment, the electrophoretic mobility of c-HSA was accelerated with increasing concentration of KCNO (Fig. 4).

### 3.5. Fluorescence characteristics of Th T bound c-HSA

**Fig. 5:**
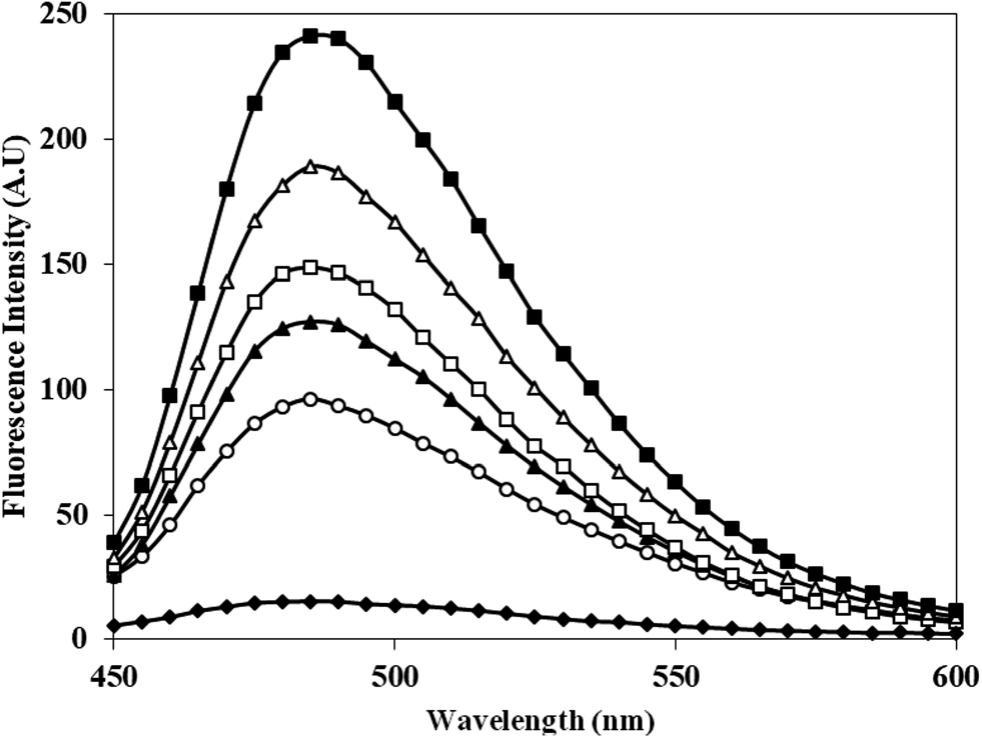
Emission profile of Th T alone (-♦-), native HSA (-○-) and at different molar ratios of HSA : KCNO, 1 : 555 (-▲-), 1 : 1111 (-□-), 1 : 1666 (-△-) and 1 : 2222 (-■-).

Th T shows enhanced fluorescence emission at 482 nm when bound to protein aggregates. Therefore, aggregate formation in c-HSA was studied by Th T binding. The emission intensity of Th T increased when mixed with c-HSA (Fig. 5). However, under identical conditions Th T binding with native HSA was almost negligible. The results suggest presence of aggregate(s) in c-HSA.

### 3.6. Carbonyl content

**Fig. 6:**
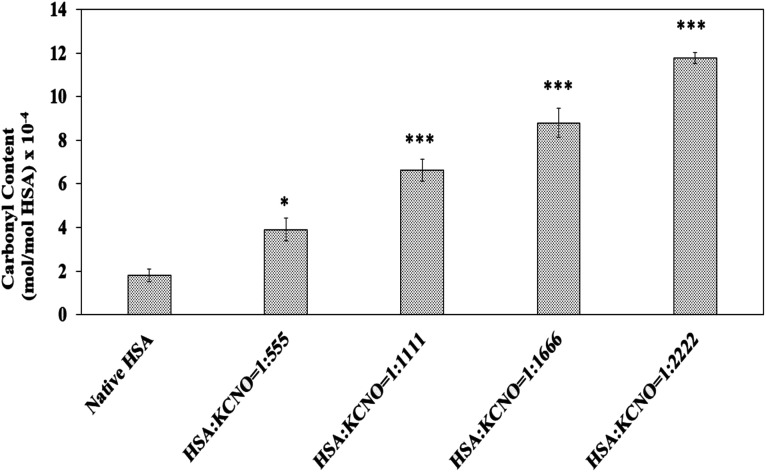
Carbonyl content of native HSA and c-HSA samples. Each bar represents mean ± SD of 3 independent assays (*p < 0.05, **p < 0.005 and ***p < 0.0005 are significantly different from native).

Dinitrophenyl hydrazine reactive carbonyls were measured to address whether carbamylation of albumin has produced oxidative stress in the system and the results are shown in Fig. 6. The carbonyl content of native HSA was found to be 1.8 × 10^−4^ mol mol^−1^ of HSA. Modification of HSA by potassium cyanate caused increase in carbonyl content as compared to native HSA. The level of carbonyl generated were different in 04 versions of c-HSA and was found to be 4.0, 6.7, 8.8, 11.7 (×10^−4^ mol mol^−1^) of HSA, respectively at 1 : 555, 1 : 1111, 1 : 1666 and 1 : 2222 molar ratios of HSA : KCNO.

### 3.7. Estimation of lysine and arginine in carbamylated-HSA

**Fig. 7:**
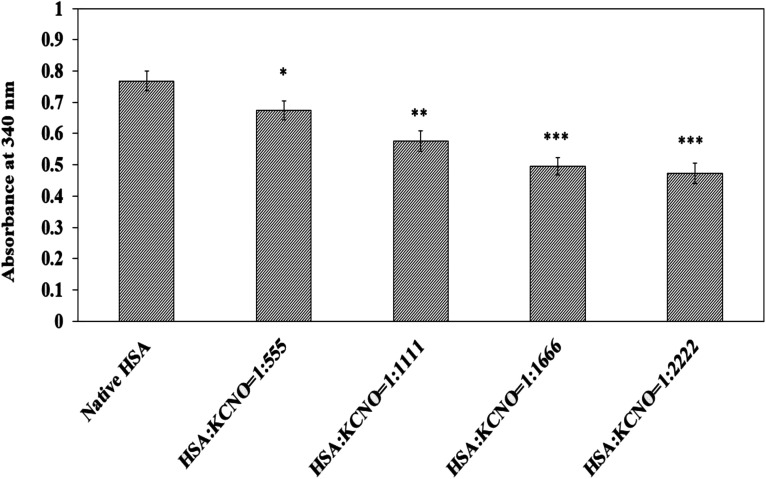
Lysine estimation in native and c-HSA samples by TNBS reagent. Each bar represents mean ± SD of 3 independent assays (*p < 0.05, **p < 0.005 and ***p < 0.0005 are significantly different from native).

The carbamyl moiety released from potassium cyanate may react and bind with lysine and arginine residues which are prone to carbamylation. The available lysine residues in native and c-HSA samples after reaction with TNBS are shown in Fig. 7. The results suggest engagement of lysine residues by the carbamyl group. A total of 41.17 percent of lysine residues were found to be carbamylated at 1 : 2222 molar ratio of HSA : KCNO.

**Fig. 8:**
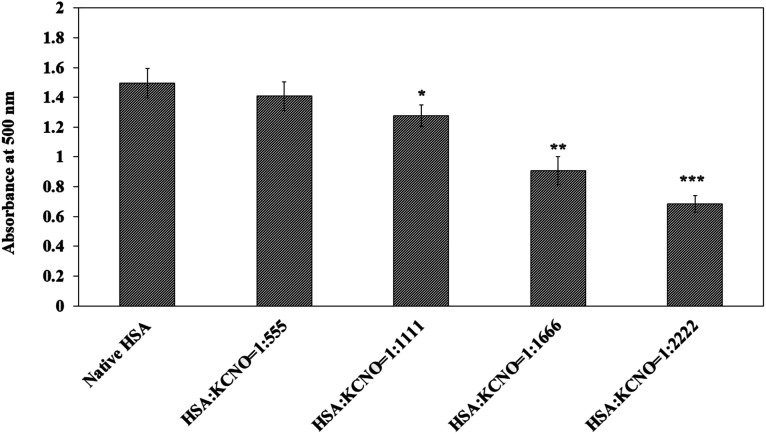
Estimation of arginine in native and c-HSA samples by Sakaguchi method. Each bar represents mean ± SD of 3 independent assays (*p < 0.05, **p < 0.005 and ***p < 0.0005 are significantly different from native).

Similarly, the reaction of carbamyl with arginine was studied with the help of Sakaguchi reaction and the results are shown in Fig. 8. Approximately 40 percent of arginine residues were bound when the molar ratio of HSA : KCNO was 1 : 2222.

### 3.8. Matrix-assisted laser desorption ionization-time of flight (MALDI-TOF) of c-HSA

**Fig. 9:**
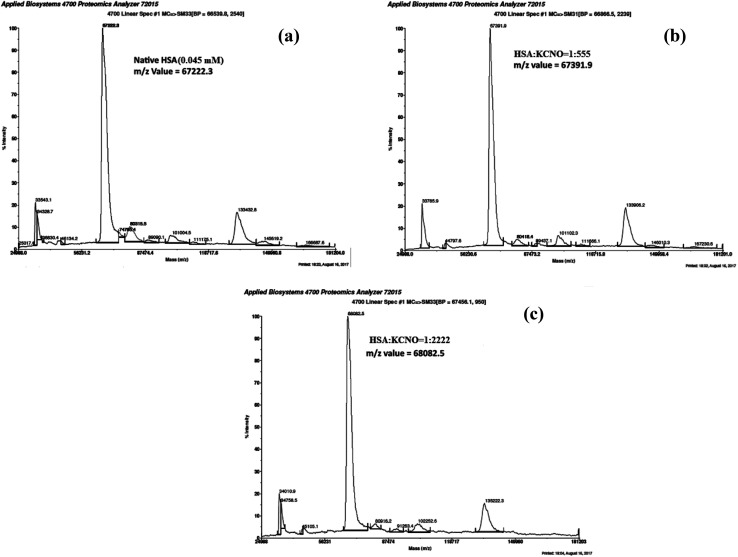
MALDI-TOF mass spectra of native HSA (a) and at different molar ratios of HSA : KCNO; 1 : 555 (b) 1 : 2222 (c).

MALDI-TOF mass spectrometry was employed to determine the mass of c-HSA. The *m*/*z* value for native HSA was found to be 67 222.3 Da (Fig. 9a). Under identical conditions the *m*/*z* value for c-HSA at HSA : KCNO molar ratio of 1 : 555 was found to be 67 391.9 Da (Fig. 9b) while at HSA : KCNO molar ratio of 1 : 2222 the mass of c-HSA was found to be 68 082.5 Da (Fig. 9c). The difference in mass of c-HSA and native HSA clearly indicates attachment of carbamyl moieties.

### 3.9. Bilirubin interaction with c-HSA

**Fig. 10:**
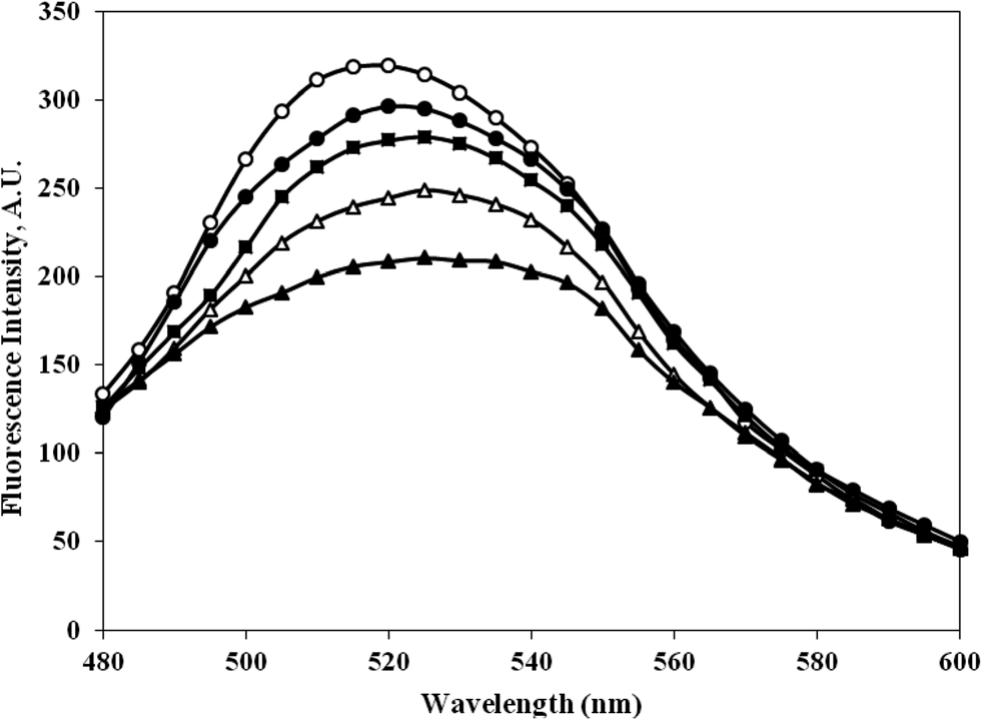
Emission profile of bilirubin mixed with native HSA (-○-) and at different molar ratios of HSA : KCNO, 1 : 555 (-●-), 1 : 1111 (-■-), 1 : 1666 (-△-) and 1 : 2222 (-▲-).

The bilirubin binding capacity of c-HSA was significantly decreased as compared to native HSA (Fig. 10). Furthermore, there was inverse relationship between bilirubin binding and albumin carbamylation. The bilirubin dianions reversibly combines with HSA in alkaline solution.

### 3.10. Binding stoichiometry of KNCO and HSA

**Fig. 11:**
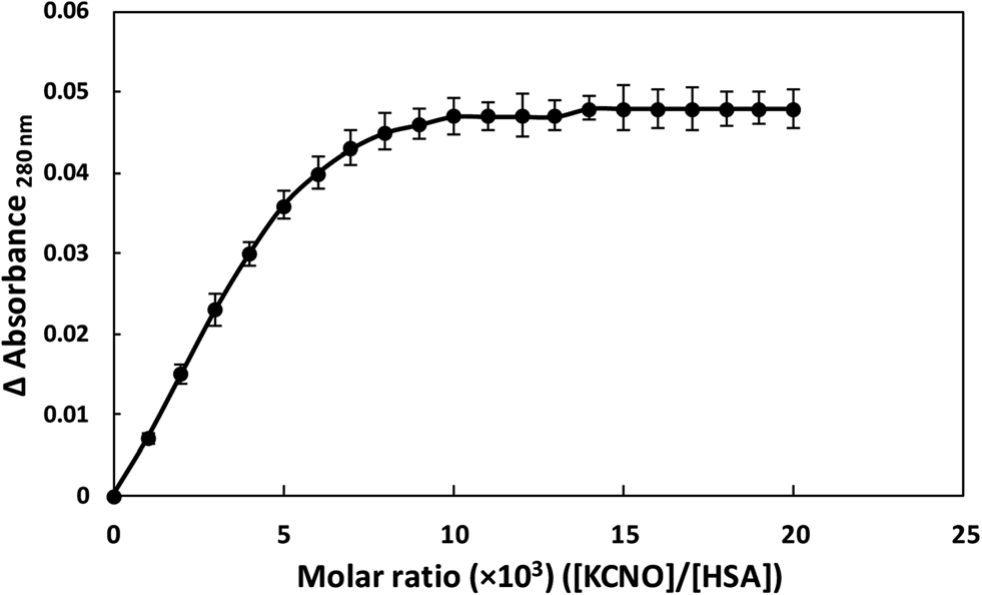
Stoichiometry of KCNO and HSA interaction.

HSA showed absorption maximum at 280 nm and stoichiometric studies were carried out at this wavelength. When HSA and KCNO were mixed in different molar ratios (1 : 1000–1 : 20 000), we observed change in absorbance upto 1 : 10 000 molar ratio of HSA and KCNO. Further increase in KCNO concentration did not produce any change in absorbance as shown in Fig. 11.

## 4. Discussion

HSA is a dynamic protein and performs an array of functions in humans. Numerous studies have demonstrated that the structure and function of albumin is affected by glucose, methylglyoxal, urea, *etc.*^23^ High urea concentration over a prolonged period results in carbamylation of various proteins present in the system which results in various pathological conditions such as CKD and ESRD.^3^ Spontaneous non-enzymatic modification of proteins can act as endogenous toxins and could be involved in a number of pathological conditions.^3^ The higher level of carbamylation could be closely related to smoking and various inflammatory diseases.^3^ This could be a possible mechanism that can contribute to various pathological conditions.^3^

In the present study, potassium cyanate (source of carbamyl group) caused carbamylation on albumin that showed hypochromicity at 280 nm. The reason being decreased accessibility of ultraviolet light on chromophoric residues (mainly tyrosine and tryptophan) due to carbamylation.^24,25^ Furthermore, the fluorescence measurement gives a vital information about the molecular environment in and around the fluorophore molecules. Therefore, conformational changes in the human serum albumin was evaluated by studying the intrinsic fluorescence.^26^ The ligand (KCNO) quenches the fluorescence intensity of HSA due to changes in the microenvironment around tryptophan residues. On increasing the concentration of KCNO, the fluorescence intensity decreased due to a variety of molecular interactions, *viz.*, excited-state reactions, molecular rearrangements, ground state complex formation and collisional quenching.^27^ The evidence regarding alterations in the secondary structure of carbamylated-HSA was collected from FT-IR results. The infrared spectra of proteins exhibit a number of amide bands due to different vibrations of the peptide moiety. Of all the amide bonds in proteins, amide I is more sensitive to the changes in protein secondary structure than amide II.^28^ The decrease in the transmittance and shift in peak position at amide I and amide II band indicated the changes in secondary structure of HSA induced by carbamylation.^29^ The saturation point existed approximately at 1 : 10 000 molar ratio of HSA to KCNO. The result showed that the HSA : KCNO molar ratios used in our study *i.e.* 1 : 555 to 1 : 2222 was not saturating. It clearly reveals that there is a clear stoichiometry of reduction of KCNO to HSA as a plateau is observed. To saturate 0.045 mM HSA used in our study, approximately 450 mM of KCNO will be required based on the stoichiometry result.

The results of FT-IR were further confirmed by far UV-CD study, which also suggested changes in the secondary structure of c-HSA. CD spectroscopy is a powerful tool to study proteins secondary structures. Reduction in alpha helicity of c-HSA sample signifies perturbations in secondary structures. The observed reduction in alpha helicity is a clear indication of accelerated peptide aggregation and/or crosslinking as depicted by enhanced Th T fluorescence.^30^

Moreover, the alteration of electrophoretic mobility with increasing potassium cyanate concentration confirms the modification of protein charge by carbamylation.^31^ The attachment of carbamyl group with HSA has been clearly shown by increase in molecular mass of c-HSA as revealed by the MALDI-TOF studies.^18^ Moreover, HSA treated at 1 : 2222 molar ratio of HSA : KCNO showed greater *m*/*z* value compared to the HSA sample treated at 1 : 555 molar ratio of HSA : KCNO. This suggests that carbamylation is a concentration dependent process that also corroborated with previous findings.^32^ Also, the findings indicate that the structural changes induced by carbamylation could affect the structural integrity of proteins.

Biochemically, oxidative stress is assessed as carbonyl which are quite stable. Under the given experimental conditions, the carbonyl content was higher in c-HSA as compared to the native HSA, suggesting oxidation of amino acid residues (lysine, arginine, cysteine *etc.*). This may affect biological as well as transport function of c-HSA.^13^

The functional properties or transport properties of c-HSA was also studied in presence of bilirubin. Bilirubin binds HSA in a ratio of 2 : 1. The bilirubin dianions reversibly combines with HSA in alkaline solution.^21^ The fluorescence data collected on bilirubin interaction with native and c-HSA suggests that c-HSA capacity to bind bilirubin has decreased.^22^.

## 5. Conclusion

We conclude that carbamylation has profound impact on the structure and function of HSA. Therefore, pathophysiological consequences of protein carbamylation may represent a new modifiable risk factor for various clinical complications. In addition, carbamylation being an adverse event for protein structure and function, we hypothesize that mechanisms involved in the prevention of protein carbamylation have to be further addressed for better understanding of molecular mechanisms involved in the etiology of diseases like CKD, thus providing an attractive target for future research.

## Funding

This work was partially funded by the Indian Council of Medical Research through grant no. 61/01/2011-BMS.

## Conflicts of interest

The authors declare no conflict of interest, financial or otherwise.

## Abbreviations

HSA: Human serum albumin
c-HSA: Carbamylated human serum albumin
CKD: Chronic kidney disease
ROS: Reactive oxygen species
CDPs: Carbamylation derived products
KCNO: Potassium cyanate

## Supplementary Material
